# Management Strategies and Outcomes for Hyponatremia in Cirrhosis in the Hyponatremia Registry

**DOI:** 10.1155/2018/1579508

**Published:** 2018-09-27

**Authors:** Samuel H. Sigal, Alpesh Amin, Joseph A. Chiodo, Arun Sanyal

**Affiliations:** ^1^Department of Medicine, Montefiore Medical Center and Albert Einstein College of Medicine, Bronx, New York 10467, USA; ^2^Department of Medicine, University of California, Irvine, California 92868, USA; ^3^Agile Therapeutics, Inc., Princeton, New Jersey 08540, USA; ^4^Virginia Commonwealth University Medical Center, Richmond, Virginia 23298, USA

## Abstract

**Aim:**

Treatment practices and effectiveness in cirrhotic patients with hyponatremia (HN) in the HN Registry were assessed.

**Methods:**

Characteristics, treatments, and outcomes were compared between patients with HN at admission and during hospitalization. For HN at admission, serum sodium concentration [Na] response was analyzed until correction to > 130 mmol/L, switch to secondary therapy, or discharge or death with sodium ≤ 130 mmol/L.

**Results:**

Patients with HN at admission had a lower [Na] and shorter length of stay (LOS) than those who developed HN (*P* < 0.001). Most common initial treatments were isotonic saline (NS, 36%), fluid restriction (FR, 33%), and no specific therapy (NST, 20%). Baseline [Na] was higher in patients treated with NST, FR, or NS versus hypertonic saline (HS) and tolvaptan (Tol) (*P* < 0.05). Treatment success occurred in 39%, 39%, 52%, 78%, and 81% of patients with NST, FR, NS, HS, and Tol, respectively. Relapse occurred in 55% after correction and was associated with increased LOS (9 versus 6 days,* P* < 0.001). 34% admitted with HN were discharged with HN corrected.

**Conclusions:**

Treatment approaches for HN were variable and frequently ineffective. Success was greatest with HS and Tol. Relapse of HN is associated with increased LOS.

## 1. Introduction

Dilutional hyponatremia (HN) is a frequent consequence of severe portal hypertension in cirrhosis. It is the result of severe vasodilation, leading to increased arginine vasopressin (AVP) release and consequent water retention [1, 2]. HN is especially common in the hospitalized patient [3] and is associated with severe ascites, hepatic encephalopathy (HE), and impaired renal function. In a retrospective study of 20,000 patients, HN was predictive of worsening disease, mortality, a higher 30-day readmission rate [4], and a 1.74-day increase in average hospital length of stay (LOS).

Management options for HN include discontinuation of diuretics, fluid restriction (FR), and administration of isotonic saline (NS), hypertonic saline (HS), or a vasopressin-receptor antagonist (or “vaptan”). FR is usually the first treatment used but is limited by patient adherence. Administration of NS and HS is problematic as they exacerbate fluid overload and ascites. Vaptans block the actions of AVP at vasopressin-2 receptors in cells of the renal collecting duct and provide a targeted approach to treatment in patients with inappropriately elevated AVP levels [5, 6]. There are currently two FDA-approved vaptans: conivaptan (Cumberland Pharmaceuticals, Inc., Nashville, Tennessee, USA) is a dual vasopressin-1A/2-receptor antagonist available for IV use, and tolvaptan (TO; Otsuka Pharmaceutical Co., Ltd., Tokyo, Japan) is an oral selective vasopressin-2-receptor antagonist [5, 6]. Both are indicated for euvolemic and hypervolemic HN. Cirrhosis was initially an approved indication for TO but was subsequently removed due to the development of hepatocellular injury during an investigational study of its use in autosomal dominant polycystic kidney disease [7].

The effectiveness of treatment strategies and impact on LOS for hospitalized cirrhotic patients with HN have not been previously reported. The HN Registry (NCT01240668) is an observational, multicenter, real-world study of patients hospitalized with euvolemic or hypervolemic HN. The objectives were to obtain clinical characteristics of patients and assess treatment practices, effectiveness, and resource utilization using LOS as a surrogate. The results of the entire population have previously been published [8]. In that report, the management and response of all patients with HN regardless of underlying condition were reported with only mention of the percentage of patients with cirrhosis. This analysis specifically assessed the subpopulation of patients with cirrhosis.

## 2. Materials and Methods

### 2.1. Study Design

Data from cirrhotic patients enrolled in the HN Registry [8] without concomitant nephrotic proteinuria, severe cardiomyopathy (ejection fraction <50%), or severe azotemia (creatinine ≥3.0 mg/dL) were entered in a database that included clinical characteristics, laboratory results, volume of fluid intake and output over each 24-hour period (if available), amount of FR, treatment with IV NS and HS, diuretics, medications used to treat HN, paracentesis, and LOS [8]. Severity of ascites and hepatic encephalopathy (HE) were recorded, and Child-Pugh scores, MELD, and MELD-Na scores were calculated [9]. Patients were classified as having HN diagnosed at the time of hospital admission versus hospital-acquired HN and categorized as mild ([Na^+^] > 125–130 mmol/L), moderate (120–125 mmol/L), and severe (< 120 mmol/L).

Initial treatment for HN was recorded. FR was based on an order by the treating physician. NS treatment was defined as administration of > 500 ml NS over a 24-hour period. No specified therapy (NST) was defined as observation for ≥2 days without a specific treatment. Patients who received NS, HS, or TO alone or in conjunction with FR were combined. A 1-day gap of no therapy between 2 treatment episodes constituted the end of initial treatment except for patients receiving TO in which case a 1-day gap was permitted. The study was exclusively observational, and treatment was solely determined by the treating physician.

Response to therapy for patients admitted with HN was assessed daily. On each day, patients were categorized based on HN severity and achievement of a treatment endpoint (correction to [Na^+^] > 130 mmol/L, increase in [Na^+^] ≥ 5 mmol/L from baseline, switch to another therapy, discharge with persistent HN, or death or transfer to hospice with persistent HN). The first [Na^+^] obtained on the day after treatment was discontinued and was then used as the end-of-therapy value. Patients in whom [Na^+^] corrected to > 130 mmol/L with initial therapy were assessed for relapse of HN during the subsequent hospitalization. Patients who were switched by the treating physician to a different treatment were assessed and reclassified based on second treatment provided in the same manner as that for the initial therapy.

At hospital discharge, final [Na^+^] and disposition (discharge home with corrected or persistent HN, and mortality [hospital death or transfer to hospice]) were recorded, and LOS was determined. For patients in whom discharge was delayed due to nonmedical reasons, the additional days were not included in LOS if documented as such in the clinical record.

### 2.2. Statistical Analysis

Clinical characteristics, initial and final [Na^+^], hospital mortality, and median LOS of patients with HN at hospital admission versus those who developed HN during hospitalization were compared. The relationship between HN severity and the various clinical parameters, LOS, and hospital mortality were assessed for patients admitted with HN.

Characteristics were compared among the various treatment groups. Cumulative endpoint outcomes were recorded for Days 1–5 and final outcomes at the end of primary treatment. The percentage of patients with initial [Na] < 125 in whom the level increased by ≥ 5 mmol/L on Days 2 and 3 and at the end of therapy was assessed. For patients with moderate or severe HN, the percentage of patients with an increase in [Na^+^] ≥ 5 mmol/L was assessed on Days 2 and 3 and end of treatment. A similar analysis was performed for patients who received a secondary therapy. For patients in whom [Na^+^] corrected to >130 mmol/L with initial therapy, characteristics and LOS were compared between patients who did and did not experience a relapse.

Descriptive statistics for continuous variables consisted of median number of observations and interquartile range (IQR). Frequency counts and percentages were obtained for categorical variables. Statistical comparisons of continuous variables were performed using nonparametric tests such as the Wilcoxon rank-sum test. Comparisons of categorical variables were performed using chi-square tests for association. Statistical significance for the tests was defined at the 5% level (*P* < 0.05).

### 2.3. Internal Review Board Approval

Approval was sought from the local research ethics review board at each site using either informed consent or a waiver of consent.

## 3. Results

### 3.1. Patient Characteristics

Of the 3087 patients who satisfied the inclusion and exclusion criteria, 650 (21%) had cirrhosis and 595 met the criteria for the current analysis. Baseline characteristics are presented in Table 1. HN was associated with advanced liver disease and severe portal hypertension (Table 2). HN was present in 518 patients (87%) on admission and developed during hospitalization in 77 (13%). Patients with HN on admission had lower initial [Na^+^], higher blood urea nitrogen (BUN), and MELD-Na score (*P* < 0.05; Table 2). More than half of the patients had large-volume ascites (Supplemental Table 1). Patients with moderate (25%;* P* < 0.05) or severe HN (28%;* P* < 0.05) more commonly had overt HE than those with mild HN (17%).

### 3.2. Initial HN Treatment

The most common initial therapies were NS (36%), FR (33%), NST (20%), TO (5%), and HS (2%; Supplemental Table 2). A variety of other therapies (e.g., salt tablets and conivaptan) and combinations were administered to 22 patients (4%). Initial [Na^+^] in the NST group was higher than in the other groups (*P* < 0.05). Initial [Na^+^] in the FR group was higher than in the HS and TO groups (both* P* < 0.05). [Na^+^] increased at greater rates in the FR versus NST group (*P* = 0.03), NS versus NST and FR groups (*P* < 0.05), and HS and TO versus NST and FR groups (*P* < 0.05 for all). Median length of treatment for NST, FR, and TO was 3 days and 2 days for NS and HS. It is important to note that the goal of this observational study was to demonstrate the current state of treatment management of hypervolemic HN in various real-world hospital settings. The duration of therapy and the treatment choice were determined by the treating physician as indicated in the clinical chart.

**Figure 1:**
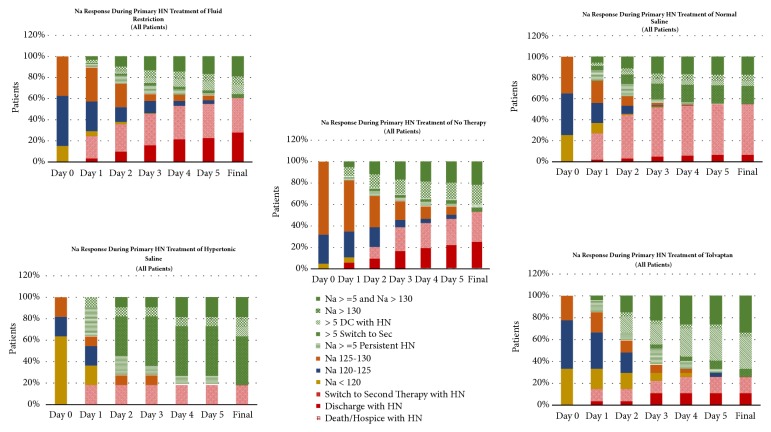
Response to primary hyponatremia (HN) treatment by various HN categories. [Na^+^], sodium concentration; FR, fluid restriction; NS, isotonic saline; NST, no specified therapy; HS, hypertonic saline; TO, tolvaptan; DC, discontinuation.

Figure 1 presents the response to initial treatment by various HN categories at Days 1–5. The percentages of patients with moderate or severe HN were significantly higher in the HS (82%) and TO (78%) groups than in the NS (65%) and FR (63%) groups, which, in turn, were higher than in the NST group (32%; all* P* < 0.05). Patients in the HS or TO groups more frequently improved into more less severe HN or treatment success categories than in those treated with NST, FR, or NS.

Table 3 presents the percentages of patients with moderate or severe HN in which [Na^+^] increased by ≥ 5 mmol/L at Days 2 and 3 and at final outcome. In the NST and FR groups, 27% of patients achieved this endpoint at Day 2 and 33% and 36%, respectively, at Day 3. Higher percentages achieved this endpoint in the NS versus NST and FR groups at Day 2 (*P* = 0.08 and = 0.02, respectively) and Day 3 (*P* = 0.08 and < 0.01). There was a more rapid response in the HS versus NST and FR groups (Days 2 and 3,* P* < 0.03 for both) and TO versus NST and FR groups (Day 2 [*P* = 0.15 and 0.07, respectively] and Day 3 [*P* < 0.01 for both]). The percentages of patients with treatment success were significantly higher in the TO versus NST, FR, and NS groups, and HS versus FR group (all* P* < 0.05).

Of patients admitted to the hospital with HN, 151 (29%) were not receiving diuretic therapy prior to hospital admission. Among these patients, 34 (23%) received ≥ 1 dose of a diuretic during initial HN therapy. Of 367 patients (71%) who received diuretics prior to hospital admission, 287 (78%) received ≥ 1 dose of a diuretic during initial HN therapy.

### 3.3. Secondary HN Treatment

A second therapy was provided to 275 patients. The secondary HN treatments based on initial therapy, and the characteristics and outcomes by secondary treatment group are presented in Supplemental Tables 4 and 5. Sodium levels prior to the secondary therapy and response rates are presented in Table 3. In general, the sodium response was similar for a specific therapy regardless of whether it was administered as initial or secondary therapy.

### 3.4. HN Relapse and Final Outcomes

Of the 110 patients who corrected with initial therapy, 61 (55%) experienced a relapse of HN during the subsequent hospitalization (Table 4). Characteristics, initial and final [Na^+^] levels, and LOS until HN correction were comparable between patients who did and did not relapse. The LOS after HN correction (6 versus 2 days) and total LOS (9 versus 6 days) were higher in patients who relapsed versus those patients whose [Na] remained above 130 (*P* < 0.05).

For patients admitted with HN, final median [Na^+^] was 129 (IQR 7) mmol/L. Of these patients, 174 (34%) were discharged alive with corrected HN, 292 (56%) were discharged with persistent HN (mild: 203 [39%]; moderate: 84 [16%]; and severe: 5 [1%]), and 49 (10%) died during hospitalization or were discharged to hospice. For patients who developed HN during hospitalization, final median [Na^+^] was 130 (IQR 6) mmol/L. Of these patients, 25 (33%) were discharged alive with corrected HN, 39 (51%) were discharged with persistent HN (mild: 31 [40%]; moderate: 8 [10%]; and severe: 0), and 12 (16%) died during hospitalization or were discharged to hospice.

**Figure 2:**
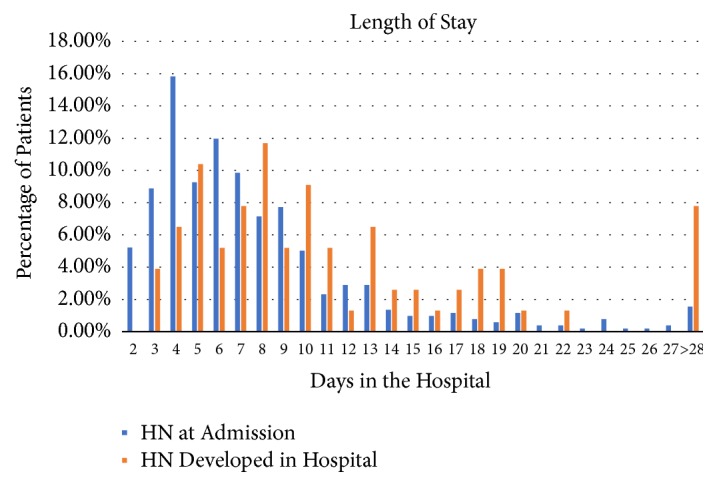
The distribution of length of stay (LOS) for patients with hyponatremia (HN) at admission versus those who developed HN during hospitalization.

Median (IQR) LOS values for patients admitted with HN and those who developed HN during hospitalization were 6 (5) and 9 (8), respectively (*P* < 0.05). The distribution of LOS for patients admitted with HN versus those who developed HN during hospitalization is presented in Figure 2. Of patients admitted with HN compared with those who developed HN during hospitalization, 90% versus 75% were discharged by 14 days. Hospital mortality was numerically greater in patients who developed HN during hospitalization than in those admitted with HN but not statistically significant (16% versus 10%;* P* = 0.25).

## 4. Discussion

This analysis of the HN Registry—the largest observational study to specifically examine HN in the hospital setting—produced several important findings. There was a strong association between HN and advanced cirrhosis with severe portal hypertension as has been previously reported [3]. Large-volume ascites and overt HE were noted in 54% and 22% of patients admitted with HN, respectively. There was not a relationship between the presence of severe ascites and the severity of HN (p = 0.216). However, the prevalence of overt HE was related to HN severity.

Treatment approaches were highly variable and frequently ineffective [7]. Twenty percent of patients received NST, and only 34% of the cirrhotic patients admitted to the hospital with HN were discharged with corrected HN. Although FR is recommended in treatment guidelines as initial therapy [10], this is the first study to report outcomes in patients treated with FR in real life practice. The rate of increase in [Na^+^] with FR treatment was of limited efficacy and comparable to NST. Of patients with moderate and severe HN, [Na^+^] increased by ≥ 5 mmol/L in only 27% and 36% at Days 2 and 3, respectively. These results are in accordance with previous studies that showed a limited or no response with FR [11, 12].

Although not recommended, treatment with NS was the most common initial therapy [13]. Ascites is frequently the reason for hospitalization, and NS can exacerbate its severity and increase the need for invasive procedures, such as paracentesis. It may also exacerbate HN through “a desalination process” in which increased AVP levels lead to water retention and excretion of hypertonic urine [14]. Patients receiving NS had lower [Na^+^] than those receiving FR. Although the rate of increase in [Na^+^] was greater, its effectiveness was limited: of patients with moderate or severe HN, [Na^+^] only increased by ≥ 5 mmol/L in 45% and 51% of patients at Days 2 and 3, respectively.

Correction of HN was most effective with HS and TO. HS is recommended for severe symptomatic HN and was used as initial therapy in 2% of patients [15]. [Na^+^] was lowest in patients receiving HS, and 82% had either moderate or severe HN. There are currently no reports on the response to HS in cirrhosis. In this study, HS led to a rapid increase in [Na^+^]—an increase ≥ 5 mmol/L by Day 2 in 78% of patients.

TO was used as primary therapy in 5% of patients. The severity of HN was similar to those receiving HS, and 78% patients had either moderate or severe HN. As with HS, [Na^+^] rapidly increased but at a slightly more gradual rate. At Day 2, 48% of patients had an increase ≥ 5 mmol/L. However, the percentage at Day 3 was comparable to HS (71% versus 78%;* P* = 1.00). This response rate was comparable to that observed in the cirrhosis population from the SALT1/SALT2 trials in which 40% and 70% of patients treated with TO had an increase ≥ 5 mmol/L at Days 2 and 3, respectively (data on file).

HN is frequent in patients with large-volume ascites [3]. In addition to the nonosmotic release of AVP, the renin-aldosterone system is activated, leading to increased renal sodium reabsorption. Diuretics are the mainstay of treatment of fluid overload but can exacerbate HN by decreasing intravascular volume (leading to increased AVP release) and only blocking sodium reabsorption, leaving continued free-water absorption unopposed [16]. Guidelines recommend tapering and then discontinuing diuretics if HN persists despite FR. However, diuretics were discontinued in only 17% of patients with HN at admission and were initiated in 7% of patients.

HN is associated with increased LOS in hospitalized patients [17–19]. In this study the impact of HN relapse in LOS after initial sodium correction was especially striking. Despite comparable initial Na level, level at correction, and time to correction, LOS was 4 days longer in those in whom HN recurred.

The results of this study are limited by its observational nature and broad definitions. FR was defined only by the physician orders indicated in the chart. NS and diuretic administration were also broadly defined. Albumin administration which is increasingly being used for the treatment of HN and infectious complications were not assessed [20]. In addition, Na levels have recently been reported to increase in response to treatment with midodrine and octreotide in a noncontrolled study [21]. However, the goal of this study was to evaluate treatment practices and outcomes in the real-world setting with the most commonly used approaches. The frequent administration of ineffective and/or nonstandardized therapy that frequently includes NS is not consistent with treatment guidelines. Correction of only 34% of patients at discharge suggests that most physicians do not view correction of HN as a meaningful clinical endpoint. Correction of HN is less important than demonstration of a beneficial impact on clinical endpoints. A question that invariably arises is whether HN is a direct participant in the pathophysiologic process and directly contributes to poor outcomes and increased LOS, or whether it is only a marker of end-stage disease. Preliminary evidence supports a contributory role for HN in hepatic encephalopathy [22]. Finally, treatment of HN with TO has also been shown to shorten LOS in patients with heart failure, syndrome of inappropriate diuretic hormone, and cancer [23, 24].

Determination of the impact of the treatment of HN in patients with cirrhosis will first require standardization of its management with effective therapy. The initial use of FR should be reevaluated, while NS administration should be avoided. Although HS is effective, it is limited by its deleterious impact on fluid overload and need for close monitoring, which frequently requires an intensive-care setting. In addition, the development of tense ascites due to HS administration can aggravate the severity of portal hypertension [23, 24]. Early treatment with TO offers an effective approach that should allow comprehensive assessment of the importance of HN treatment in the hospitalized patient with cirrhosis. Because the FDA-approved indications for TO have removed cirrhosis as an approved population, it is important that treatment with TO in these patients be performed in a carefully controlled manner.

## Figures and Tables

**Table 1:** Baseline demographic characteristics.

	All Patients^i^ (N = 3,087)	Cirrhosis (n = 630)
Age distribution, n (%)^a^		
≤50 y	479 (16)	190 (30)
51–64 y	937 (30)	339 (54)
65–74 y	587 (19)	81 (13)
≥75 y	1,084 (35)	20 (3)
Men, n (%)^b^	1,558 (51)	419 (67)

Race distribution: US only, n (%)^a^		
White	1,927 (74)	455 (72)
African-American	309 (12)	58 (9)
Asian	57 (2)	13 (2)
Other	154 (6)	53 (9)
Unknown	149 (6)	51 (8)
Mean initial [Na+] ± SD, mEq/L^c^	123.6 ± 5.5	124.1 ± 5.0
Mean initial BUN ± SD, mg/dL^a^	20.8 ± 16.8	25.5 ± 18.8
Mean initial creatinine ± SD, mg/dL^d^	1.1 ± 0.73	1.28 ± 0.85
Initial BUN:creatinine ratio^a^	19.4 ± 9.4	19.8 ± 8.6

Prior HN, n (%)^a,e^		
Yes	909 (29)	240 (38)
No	1,176 (38)	178 (28)
Unknown	1,001 (32)	212 (34)

HN at admission, n (%)^f^		
Yes	2,532 (82)	549 (87)
No	531 (17)	81 (13)
Unknown	24 (1)	0 (0)

Primary physician specialty, n (%)		
Nephrologist	104 (3)	8 (1)
Endocrinologist	108 (4)	0
Cardiologist	321 (10)	7 (1)
Hepatologist	260 (8)	246 (39)
Oncologist	111 (4)	11 (2)
Generalist	1,844 (60)	315 (50)
Other	338 (11)	43 (7)

HN subspecialist consulted, n (%)^g,h^		
No	1989 (64)	501 (80)
Yes	1,096 (36)	129 (21)

Abbreviations: BUN, blood urea nitrogen; CHF, congestive heart failure; HN, hyponatremia; [Na^+^], sodium concentration; SD, standard deviation; SIADH, syndrome of inappropriate antidiuretic hormone secretion.

^a^SIADH vs CHF and cirrhosis, and CHF vs cirrhosis: *P *<0.001.

^b^SIADH vs CHF: *P *= 0.79; and SIADH and CHF vs cirrhosis: *P *<0.001.

^c^SIADH vs CHF and cirrhosis: *P *<0.001; CHF vs cirrhosis: *P *= 0.01.

^d^SIADH vs CHF and cirrhosis: *P *<0.001; and CHF vs cirrhosis: *P *= 0.05.

^e^HN during previous hospital admission in prior 12 months.

^f^Data missing for 24 patients in All, 19 in SIADH, and 4 in CHF populations; SIADH vs CHF: *P *= 0.04; SIADH vs cirrhosis: *P *= 0.001; and CHF vs cirrhosis: *P *<0.001.

^g^SIADH vs CHF and cirrhosis: *P *<0.001; and CHF vs cirrhosis: *P *= 0.01.

^h^HN specialist defined as nephrologist or endocrinologist.

^i^Includes 171 patients without a diagnosis of SIADH, cirrhosis, or CHF.

**Table 2:** Clinical characteristics of cirrhosis patients admitted with HN subdivided by hyponatremia severity.

	Total N = 518	[Na^+^], mmol/L		
		<120 n = 106	≥120–≤125 n = 202	>125–≤130 n = 210
Median age, y	56	54	56	57
Male/female, n	345/173	73/33	130/72	142/68
BUN, mg/dL	20.0 (19.0)	18.0 (20.5)	21.0 (20.0)	19.0 (17.0)
Cr, mg/dL	1.0 (0.6)	1.0 (0.7)	1.1 (0.7)	1.0 (0.6)
BUN:Cr ratio	18.9 (10.4)	19.0 (12.0)	19.8 (11.6)	18.0 (9.1)
Alb, g/dL	2.5 (0.8)	2.6 (1.1)	2.5 (0.8)	2.4 (0.6)
Tbili, *µ*mol/L	4.3 (7.4)	4.3 (7.2)	4.5 (6.9)	4.3 (7.6)
INR, s	1.7 (0.6)	1.6 (0.5)	1.7 (0.7)	1.7 (0.7)
Severe ascites, n (%)	284 (55)	53 (50)	124 (61)	107 (51)
Severe HE, n (%)^a^	116 (22)	30 (28)	51 (25)	35 (17)
C-P score	11.0 (3.0)	10.5 (3.0)	11.0 (3.0)	10.0 (3.0)
MELD score	20.2 (9.7)	18.7 (9.0)	20.8 (8.0)	20.2 (10.4)
MELD-Na score	27.3 (6.3)	26.7 (5.6)	28.0 (5.0)	26.3 (7.6)

^a^ *P* < 0.01.

Values for blood urea nitrogen (BUN), creatinine (Cr), BUN:Cr ratio, albumin (Alb), total bilirubin (Tbili), international normalized ratio (INR), and Child-Pugh (CP), Model for End-Stage Liver Disease (MELD), and MELD-NA scores are median (interquartile range). HE, hepatic encephalopathy; HN, hyponatremia.

**Table 3:** Patients with [Na^+^] ≥5 mmol/L in response to initial and secondary therapy^a^.

	Day 2 Response	Day 3 response	Final response
Initial therapy, n (%)			
NST	9 (27)	11 (33)	13 (39)
FR	29 (27)	38 (36)	42 (39)
NS	54 (45)	61 (51)	62 (52)
HS	7 (78)	7 (78)	7 (78)
TO	10 (48)	15 (71)	17 (81)

Secondary therapy, n (%)			
NST	5 (36)	5 (36)	5 (36)
FR	15 (31)	18 (37)	18 (37)
NS	9 (29)	10 (32)	10 (32)
HS	10 (100)	10 (100)	10 (100)
TO	15 (58)	17 (65)	18 (68)

^a^Patients with initial moderate or severe hyponatremia.

Initial therapy* P *<0.05 for *Day 2 response*: no specific therapy (NST) vs hypertonic saline (HS), and fluid restriction (FR) vs HS and isotonic saline (NS); *Day 3 response: *NST vs HS and tolvaptan (TO); FR vs HS, NS, and TO: *final response:* NST vs TO, FR vs HS and TO, and NS vs TO.

**Table 4:** Patient characteristics and LOS for patients admitted with HN who corrected with initial HN therapy.

	Relapse (n = 61)	No relapse (n = 49)
[Na^+^], mmol/L	128.0 (4.0)	127.0 (5.0)
[Na^+^] at time of correction, mmol/L^a^	132.0 (2.0)	132.0 (2.0)
BUN, mg/dL	17.0 (17.0)	16.0 (16.0)
Cr, mg/dL	1.0 (0.5)	1.0 (0.9)
BUN:Cr ratio	19.1 (9.6)	17.4 (11.4)
Tbili, *µ*mol/L	4.8 (7.3)	4.3 (6.7)
INR, s	1.8 (0.8)	1.8 (0.5)
Alb, g/doll	2.6 (0.9)	2.8 (0.7)
MELD score at correction	20.9 (6.6)	21.9 (11.3)
LOS, d^a^	9 (6.0)	6 (5.0)
LOS until correction, d	3 (2.0)	3 (1.0)
LOS after correction, d^a^	6 (5.0)	2 (3.0)
Death/ hospice, n (%)	5 (8)	5 (10)

^a^ < 0.05.

Values for sodium concentration ([Na^+^]), [Na^+^] at time of correction, blood urea nitrogen (BUN), creatinine (Cr), BUN:CR ratio, total bilirubin (Tbili), albumin (Alb), international normalized ratio (INR), Model for End-Stage Liver Disease (MELD) score at correction, length of stay (LOS), LOS until correction, and LOS after correction are median (interquartile range). HN, hyponatremia.

## Data Availability

This was an observational study, not a randomized, controlled clinical trial, supported by Otsuka America Pharmaceutical, Inc. All statistical analyses were performed by an outside organization, Mapi Group, using predefined definitions and independent of Otsuka. In the event there were issues, they were adjudicated by 2 members of the Hyponatrema Registry Steering Committee. We would be happy to answer any additional questions. As indicated in the Methods section, all statistical analyses were performed by MAPI using predefined criteria. MAPI is an independent biostatistical organization that is routinely employed for independent analysis of data sets. All analyses were performed independently of investigator in-put. The data for each patient is present in an elaborate Excel spreadsheet that requires detailed explanation in its use.

## Supplementary Materials

Supplementary Materials Supplemental Table 1: this table provides the clinical characteristics, LOS, and mortality of cirrhotic patients in the Hyponatremia Registry. It includes a breakdown between those who presented with HN at hospital admission versus those who developed HN during the hospital stay. Supplemental Table 2: this table shows the clinical characteristics including HN severity of the cirrhotic patients with HN at admission based on initial therapy selected by the treating physician. Supplemental Table 3: this table presents the outcomes of the initial therapies of cirrhotic patients admitted with HN, including the percentages switched to a second therapy, discharged with persistent HN, mortality, and length of stay. Supplemental Table 4: this table provides the outcomes of the secondary therapies of the patients who presented with HN and did not respond to the initial therapy. The initial treatment is indicated in the far-left column and the secondary therapy is indicated in the top row. Supplemental Table 5: this table presents the outcomes of the secondary therapies of cirrhotic patients who were admitted with HN and did not respond to the initial therapy.
