# Supplementary material for: Management Strategies and Outcomes for Hyponatremia in Cirrhosis in the Hyponatremia Registry
Source: Can J Gastroenterol Hepatol. 2018 Sep 27;2018:1579508. doi: 10.1155/2018/1579508 (PMC6180997; doi:10.1155/2018/1579508)
Supplement: Supplementary Materials — Supplemental Table 1: this table provides the clinical characteristics, LOS, and mortality of cirrhotic patients in the Hyponatremia Registry. It includes a breakdown between those who presented with HN at hospital admission versus those who developed HN during the hospital stay. Supplemental Table 2: this table shows the clinical characteristics including HN severity of the cirrhotic patients with HN at admission based on initial therapy selected by the treating physician. Supplemental Table 3: this table presents the outcomes of the initial therapies of cirrhotic patients admitted with HN, including the percentages switched to a second therapy, discharged with persistent HN, mortality, and length of stay. Supplemental Table 4: this table provides the outcomes of the secondary therapies of the patients who presented with HN and did not respond to the initial therapy. The initial treatment is indicated in the far-left column and the secondary therapy is indicated in the top row. Supplemental Table 5: this table presents the outcomes of the secondary therapies of cirrhotic patients who were admitted with HN and did not respond to the initial therapy. [file 1579508.f1.pdf]

**Supplemental Table 1** Clinical characteristics of all cirrhosis patients, including LOS and hospital mortality, based on HN developing in the hospital *vs* admitted to the hospital with HN.

| | Total (n = 595) | At admission (n = 518) | In-hospital (n = 77) |
|---|---|---|---|
| [$Na^+$], mmol/L[a] | 125.0 (7.0) | 124.0 (7.0) | 129.0 (2.0) |
| BUN, mg/dL[b] | 19.0 (17.0) | 20.0 (19.0) | 16.0 (14.0) |
| Cr, mg/dL | 1.0 (0.6) | 1.0 (0.6) | 0.9 (0.4) |
| BUN:Cr ratio | 18.5 (10.4) | 18.9 (10.4) | 16.6 (10.7) |
| Tbili, µmol/L | 4.3 (7.5) | 4.3 (7.4) | 4.3 (8.6) |
| Alb, g/dL | 2.5 (0.8) | 2.5 (0.8) | 2.4 (0.8) |
| INR, s | 1.7 (0.6) | 1.7 (0.6) | 1.6 (0.7) |
| Severe Ascites, n (%)[c] | 321 (54) | 284 (55) | 37 (48) |
| Severe HE, n (%)[c] | 132 (22) | 116 (22) | 16 (21) |
| C-P Score | 11.0 (3.0) | 11.0 (3) | 10.0 (3.0) |
| MELD Score | 20.1 (9.6) | 20.2 (9.7) | 18.9 (8.2) |
| MELD-Na Score[b] | 26.9 (6.4) | 27.3 (6.3) | 25.2 (5.8) |
| LOS, d[a] | 7.0 | 6.0 | 9.0 |
| Death/Hospice n (%) | 61 (10) | 49 (10) | 12 (16) |

[a]$P$ <0.001; [b]$P$ = 0.01 for hyponatremia (HN) at admission vs developing HN in hospital.

[c]Patients with most severe cases.

Values for length of stay (LOS) reported as median; values for sodium concentration ([$Na^+$]), blood urea nitrogen (BUN), creatinine (Cr), BUN:Cr ratio, total bilirubin (Tbili), albumin (Alb), international normalized ratio (INR), and Child-Pugh (C-P), Model for End Stage Liver Disease (MELD), and MELD-NA scores are median (interquartile range). HE, hepatic encephalopathy.

**Supplemental Table 2** Clinical characteristics of cirrhosis patients admitted with HN based on initial HN therapy.

| Therapy | NST (n = 103) | FR (n = 171) | NS (n = 184) | HS (n = 11) | TO (n = 27) |
|---|---|---|---|---|---|
| Initial [$Na^+$],mmol/ | 127.0 (4.0) | 124.0 (6.0) | 124.0 (8.0) | 116.0 (14.0) | 121.0 (7.0) |
| HN severity (mild/moderate/severe), % | 68/27/5 | 37/47/15 | 35/40/26 | 18/18/64 | 22/44/33 |
| BUN, mg/dL | 19.0 (19.0) | 19.0 (17.0) | 21.0 (20.0) | 21.0 (49.0) | 18.0 (17.0) |
| Cr, mg/dL | 1.0 (0.6) | 1.0 (0.6) | 1.1 (0.8) | 1.1 (1.4) | 1.2 (0.5) |
| BUN:Cr ratio | 18.5 (12.2) | 19.2 (8.9) | 17.9 (9.8) | 18.9 (14.4) | 18.1 (9.7) |
| Tbili, µmol/L | 3.7 (8.1) | 4.7 (6.8) | 4.2 (7.7) | 3.2 (1.8) | 7.5 (8.0) |
| Alb, g/dL | 2.4 (0.7) | 2.4 (0.6) | 2.5 (0.8) | 2.8 (0.9) | 2.7 (0.4) |
| INR, s | 1.6 (0.7) | 1.7 (0.7) | 1.6 (0.6) | 1.6 (1.0) | 1.7 (0.4) |
| Severe HE, n (%) | 16 (16) | 37 (22) | 46 (25) | 4 (36) | 6 (22) |
| Severe Ascites, n (%) | 56 (54) | 101 (59) | 91 (50) | 8 (73) | 15 (56) |
| C-P Score | 10.0 (3.0) | 11.0 (2.0) | 10.0 (3.0) | 11.0 (3.0) | 12.0 (2.0) |
| MELD Score | 20.3 (10.4) | 20.0 (8.3) | 18.9 (10.4) | 15.3 (14.0) | 21.3 (5.2) |

| | | | | | |
|---|---|---|---|---|---|
| MELD-Na Score | 26.8 (7.4) | 27.3 (6.0) | 26.7 (7.0) | 24.6 (8.8) | 28.3 (3.4) |

Values for initial sodium concentration [$Na^+$], blood urea nitrogen (BUN), creatinine (Cr), BUN:Cr ratio, total bilirubin (Tbili), albumin (Alb), international normalized ratio (INR), Child-Pugh (C-P), Model for End-Stage Liver Disease (MELD), and MELD-Na scores are median (interquartile range). $P$ <0.05 for *initial [$Na^+$]*: no specific therapy (NST) vs fluid restriction (FR), normal saline (NS), hypertonic saline (HS), and tolvaptan (TO), FR vs HS and TO, NS vs HS, and HS vs TO; *Tbili:* NST vs TO; *Alb:* FR vs TO; *INR:* FR vs NS; *severe ascites:* NST vs FR, and FR vs NS; *C-P score:* NST vs FR, FR vs NS, and NS vs TO; HE, hepatic encephalopathy; HN, hyponatremia

**Supplemental Table 3** Outcomes of cirrhosis patients admitted with HN based on initial HN therapy.

| Therapy | NST (n = 103) | FR (n = 171) | NS (n = 184) | HS (n = 11) | TO (n = 27) |
|---|---|---|---|---|---|
| Final [$Na^+$], mmol/L | 128.0 (7.0) | 127.0 (7.0) | 127.0 (7.0) | 127.0 (10.0) | 130.0 (6.0) |
| Duration, d | 3.0 (3.0) | 3.0 (3.0) | 2.0 (2.0) | 2.0 (2.0) | 3.0 (2.0) |
| Rate of [$Na^+$] change, mmol/L/d | 0.5 (1.5) | 1.0 (2.0) | 1.6 (3.3) | 3.5 (5.0) | 1.8 (1.3) |
| [$Na^+$] ≥5/<131 mmol/L, % | 7 | 13 | 22 | 54 | 37 |
| [$Na^+$] <5/>131 mmol/L, % | 18 | 8 | 6 | 9 | 4 |
| [$Na^+$] ≥5/>131 mmol/L, % | 21 | 19 | 17 | 18 | 33 |
| Total, % | 46 | 40 | 45 | 81 | 74 |
| Switch therapy, % | 29 | 34 | 51 | 18 | 15 |
| Discharge with HN, % | 24 | 30 | 8 | 0 | 7 |
| Death/Hospice, % | 1 | 0 | 0 | 0 | 0 |
| LOS, d | 7 | 6 | 6 | 8 | 5 |

Values for final [$Na^+$], duration, and rate of change are median (interquartile range). *P* <0.05 for *[$Na^+$] >130 mmol/L:* NST vs FR and NS; *[$Na^+$] ≥5 mmol/L*: NST vs NS and TO, FR vs TO, and NS vs TO; and *switch therapy:* NST vs NS, FR vs NS and TO, and NS vs TO. HN, hyponatremia; LOS, length of stay.

**Supplemental Table 4** Second therapy of patients admitted with HN based on initial HN therapy.

| First therapy | | | | | | | |
|---|---|---|---|---|---|---|---|
| Secondary therapy, n (%) | NST | FR | NS | HS | TO | Other | Total |
| NST | 0 | 11 (16) | 30 (22) | 1 (13) | 0 | 1 (5) | 43 (16) |
| FR | 21 (54) | 1 (2) | 76 (57) | 4 (50) | 5 (71) | 5 (25) | 112 (41) |
| NS | 14 (36) | 30 (45) | 7 (5) | 1 (13) | 0 | 3 (15) | 55 (20) |
| HS | 0 | 2 (3) | 5 (4) | 0 | 1 (14) | 2 (10) | 10 (4) |
| TO | 4 (10) | 18 (27) | 7 (5) | 0 | 0 | 5 (25) | 34 (12) |
| Other | 0 | 5 (7) | 9 (7) | 2 (25) | 1 (14) | 4 (20) | 21 (8) |
| Total | 39 (14) | 67 (24) | 134 (49) | 8 (3) | 7 (3) | 20 (7) | 275 (100) |
| Switched to second therapy | 39 (39) | 67 (39) | 134 (73) | 9 (73) | 7 (26) | 20 (91) | NA |

**Supplemental Table 5** **C**linical characteristics and outcomes of second therapy.

| Second therapy | NST (n = 43) | FR (n = 112) | NS (n = 55) | HS (n = 10) | TO (n = 34) |
|---|---|---|---|---|---|
| Initial $[Na^+]$, mmol/L | 127.0 (4.0) | 126.0 (7.0) | 125.0 (5.0) | 117.5 (5.0) | 124.0 (3.0) |
| HN severity (mild/moderate/severe), % | 67/30/2 | 41/38/6 | 38/47/9 | NA/30/70 | 21/62/15 |
| MELD score | 21.7 (7.9) | 20.8 (7.1) | 21.7 (9.3) | 14.8 (5.8) | 21.3 (9.6) |
| Final $[Na^+]$, mmol/L | 131.0 (4.0) | 129.0 (7.0) | 127.0 (7.0) | 127.0 (3.0) | 131.0 (7.0) |
| $[Na^+]$ >5/<131 mmol/L, % | 5 | 9 | 15 | 80 | 12 |
| $[Na^+]$ <5/>131 mmol/L, % | 26 | 8 | 19 | 0 | 3 |
| $[Na^+]$ >5/>131 mmol/L, % | 16 | 18 | 12 | 20 | 55 |
| Total, % | 47 | 35 | 46 | 100 | 70 |
| Total LOS | 7.0 (4.0) | 7.0 (5.0) | 8.0 (6.0) | 10.5 (6.0) | 8.0 (4.0) |
| LOS after second therapy | 5.0 (4.0) | 6.0 (5.0) | 5.0 (6.0) | 8.0 (6.0) | 6.0 (3.0) |

Values for initial sodium concentration ([$Na^+$]), Model for End-Stage Liver Disease (MELD) score, final [$Na^+$], total length of stay (LOS), and LOS after second therapy are median (interquartile range). *P* <0.05 for *initial [$Na^+$]:* no specific treatment (NST) vs isotonic saline (NS), hypertonic saline (HS), and tolvaptan (TO), fluid restriction (FR) vs NS, HS, and TO, NS vs HS, and HS vs TO; *final [$Na^+$]:* NST vs HS and NS, and FR vs NS; and *change from initial [$Na^+$]:* NST vs HS and TO, FR vs HS and TO, NS vs HS and TO, and HS vs TO. NA, not applicable.
